# MiR-193b regulates breast cancer cell migration and vasculogenic mimicry by targeting dimethylarginine dimethylaminohydrolase 1

**DOI:** 10.1038/s41598-017-14454-1

**Published:** 2017-10-25

**Authors:** Julie-Ann Hulin, Sara Tommasi, David Elliot, Dong Gui Hu, Benjamin C. Lewis, Arduino A. Mangoni

**Affiliations:** 1 Clinical Pharmacology, Flinders University College of Medicine and Public Health, Flinders Medical Centre, Bedford Park, South Australia Australia; 2 Flinders Centre for Innovation in Cancer, Bedford Park, South Australia Australia

## Abstract

Dimethylarginine dimethylaminohydrolase 1 (DDAH1) is responsible for metabolism of an endogenous inhibitor of nitric oxide synthase (NOS), asymmetric dimethylarginine (ADMA), which plays a key role in modulating angiogenesis. In addition to angiogenesis, tumours can establish a vascular network by forming vessel-like structures from tumour cells; a process termed vasculogenic mimicry (VM). Here, we identified over-expression of DDAH1 in aggressive MDA-MB-231, MDA-MB-453 and BT549 breast cancer cell lines when compared to normal mammary epithelial cells. DDAH1 expression was inversely correlated with the microRNA miR-193b. In DDAH1^+^ MDA-MB-231 cells, ectopic expression of miR-193b reduced DDAH1 expression and the conversion of ADMA to citrulline. In DDAH1^−^ MCF7 cells, inhibition of miR-193b elevated DDAH1 expression. Luciferase reporter assays demonstrated *DDAH1* as a direct target of miR-193b. MDA-MB-231 cells organised into tube structures in an *in vitro* assay of VM, which was significantly inhibited by DDAH1 knockdown or miR-193b expression. Mechanistically, we found miR-193b regulates cell proliferation and migration of MDA-MB-231 cells, whilst DDAH1 knockdown inhibited cell migration. These studies represent the first evidence for DDAH1 expression, regulation and function in breast cancer cells, and highlights that targeting DDAH1 expression and/or enzymatic activity may be a valid option in the treatment of aggressive breast cancers.

## Introduction

Breast cancer is the most common cancer among women and accounts for a significant proportion of cancer-related death in western countries^1^. Currently there is no gold standard therapy for breast cancer due to its highly heterogeneous nature. Whilst the majority of breast cancers are positive for estrogen receptor (ER^+^), progesterone receptor (PR^+^) and/or human epidermal growth factor receptor 2 (HER2^+^), and can thus be treated with targeted endocrine therapy^2^, a small subset of breast cancers are negative for all three receptors. These tumours, termed triple negative breast cancer (TNBC), are typically treated with a less-successful combinatorial approach of chemotherapy, radiation therapy and surgery. In addition, TNBC presents as a highly proliferative and aggressive disease with rapid growth and early metastases, resulting in significantly higher mortality rates and a reduced life expectancy when compared to other molecular subtypes^3^.

Access to a blood supply plays a central role in both local tumour growth and distant metastasis of breast cancer^4^. Intra-tumoural vascular networks formed by angiogenesis, the sprouting and extension of pre-existing blood vessels, has previously been considered the only process responsible for tumour vascularisation and blood supply. However, despite the theoretical efficacy of anti-angiogenic treatments to target this process, the benefits obtained are often modest and have not proved beneficial in regards to long-term survival^5,6^. Recently, a new tumour vascular paradigm independent of endothelial cell-mediated angiogenesis has been described. Vasculogenic mimicry (VM) describes the formation of vessel-like networks directly by the tumour cells themselves^7,8^. In contrast to vessels lined by endothelial cells, channels formed by VM are lined by tumour cells yet can still fuse to a conventional vascular network to provide an adequate blood supply for tumour growth^9^. The presence of VM networks is predictive of poor survival and increased metastatic potential through entrance of tumour cells into the vasculature^10,11^, and VM inhibition is reported to abrogate tumour development^12^. The molecular mechanisms regulating VM, and whether these overlap with classical angiogenesis, are currently not well understood. However, it has been suggested that an upregulation of angiogenesis-related genes may be involved^13^.

Nitric oxide (NO) is an important cellular signalling molecule^14^. Synthesis of NO is mediated by the family of nitric oxide synthase (NOS) enzymes through conversion of arginine to L-citrulline. The methylated arginines asymmetric dimethylarginine (ADMA) and monomethyl arginine (L-NMMA) are competitive endogenous inhibitors of all isoforms of NOS^15,16^. Dimethylarginine dimethylaminohydrolase (DDAH) is the primary enzyme involved in the metabolism of ADMA and L-NMMA^17^. Whilst two isoforms of DDAH are observed in human (DDAH1 and DDAH2), current evidence suggests DDAH1 is the critical enzyme for ADMA and L-NMMA clearance^18,19^ and is thus important for the tight regulation of NO production. NO has various functions in many processes including angiogenesis and cancer^20,21^. Specifically, endothelium-derived NO promotes angiogenesis through inhibition of apoptosis^22^ and enhancement of endothelial cell proliferation and migration^23,24^. In cancer the roles of NO are diverse, and are proposed to have dual pro- and anti-tumour effects depending on local concentration^25^. An increase in inducible NOS (iNOS) expression is documented in many solid tumours including those of the breast^26–29^. Furthermore, DDAH overexpression enhances angiogenesis in tumours with an accompanied increase in metastatic potential^30,31^. Inhibition of NO synthesis significantly suppresses angiogenesis with some beneficial effects in cancer^32,33^. These findings suggest a key role for DDAH1 in the modulation of angiogenesis of endothelial cells.

A family of small non-coding RNAs (21–25 nt) called microRNAs (miRNA or miR) have recently emerged as major post-transcriptional regulators of gene expression^34^. The post-transcriptional regulatory function of miRNAs is mediated through target mRNA degradation and/or inhibition of protein translation, promoted through their binding to miRNA target sites typically located within the 3′-untranslated region (3′UTR) of target mRNAs. Each miRNA contains a unique seed sequence corresponding to nucleotides 2–7 from its 5′ terminus which determines its target-specificity and is essential for miRNA binding. The importance of miR-193b expression in cancer has been previously documented and it has been identified as a tumour suppressor in multiple cancers and cancer cell lines from pancreatic^35^, brain^36^, prostate^37^, skin^38^ and breast origins^39^. However, to date only a handful of targets of miR-193b have been identified. Using bioinformatics algorithms we identified a miR-193b target site in the 3′UTR of human *DDAH1*. This study sought to quantify miR-193b and DDAH1 expression levels in breast cancer cell lines, to assess miR-193b-mediated regulation of *DDAH1*, and to determine the roles of miR-193b and thus DDAH1 in breast cancer VM.

## Results

### DDAH1 3′UTR contains a putative miR-193b binding site

**Figure 1:**
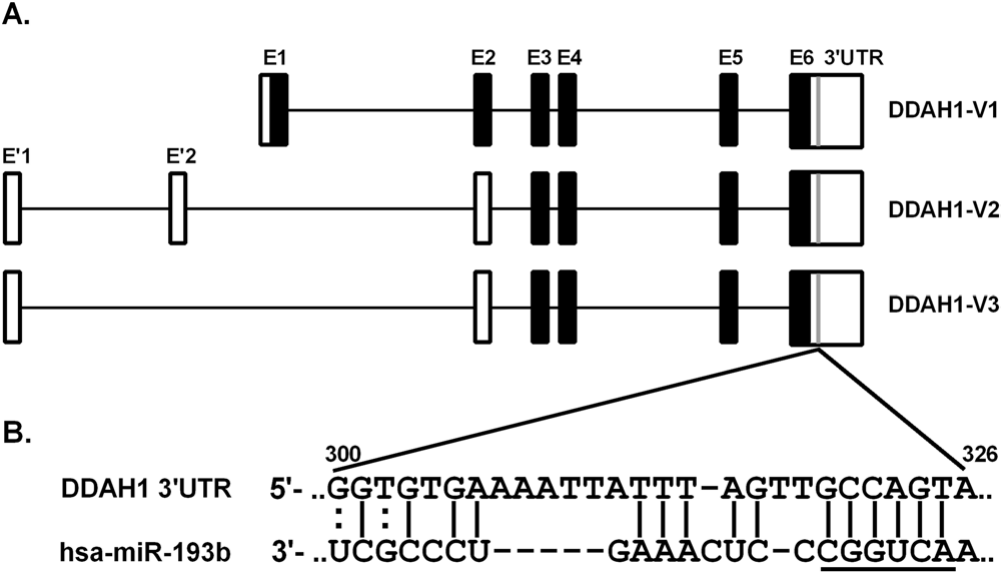
The 3′UTR of *DDAH1* mRNA contains a putative miR-193b binding site. (**A**) Gene structure and the 3 transcript variants of human *DDAH1*. Black boxes represent the open reading frame (ORF) and empty boxes represent the untranslated regions (UTRs). *DDAH1-V2* and *-V3* encode for a N-terminally truncated protein compared to *DDAH1-V1*. All 3 variants have the same 3′UTR. Grey lines represent the location of the putative miR-193b binding site. (**B**) Schematic of predicted binding of miR-193b at positions 300–326 of *DDAH1* 3′UTR. Numbering is relative to the stop codon (TGA with A positioned as −1). Underline indicates seed sequence of miR-193b. | indicates complementary base pairing and : indicates a weak binding interaction.

The *DDAH1* 3′UTR is 2,971 bp in length and thus contains numerous putative miRNA binding sites. Bioinformatic analysis target predictions with TargetScan 7.0 (www.targetscan.org), FindTar3 (bio.sz.tsinghua.edu.cn) and miRanda (microrna.org) revealed a single predicted binding site for miR-193b at position 300–326 of the *DDAH1* 3′UTR (Fig. 1A). Parameters important for efficient miRNA-mRNA hybridization such as seed pairing, extra pairing, hybridization stability and free-energy were taken into account. The predicted pairing of miR-193b to the *DDAH1* 3′UTR was given a context+ + score of −0.19 and a context+ + score percentile of 81 by TargetScan, placing the binding site in the top 19% of all predicted miR-193b sites. MiRanda ranked the putative binding site with a mirSVR score of −0.0934, an empirical probability of target inhibition, corresponding to the top 7% of predictions for miR-193b^40^. Mismatches present in the central region of the miRNA-mRNA duplex, as well as complementarity to the miRNA 3′ half (particularly at nucleotides 13–16), provides further stabilization for the interaction and enhances the efficacy of miRNA targeting^41^. Indeed, 3′-pairing to the predicted target site in the *DDAH1* 3′UTR was found at nucleotides 12–14 and 16–17 of miR-193b (Fig. 1B). The predicted binding site for the miR-193b seed sequence is fully conserved between human, chimpanzee and dog, and has a single nucleotide mismatch in rhesus, pig, cow, cat and elephant (data not shown). In summary, miR-193b exhibited a favourable predicted binding score within the *DDAH1* 3′UTR, at close proximity to the poly-A tail.

### DDAH1 is expressed in breast cancer cell lines and inversely correlates with expression of miR-193b

**Figure 2:**
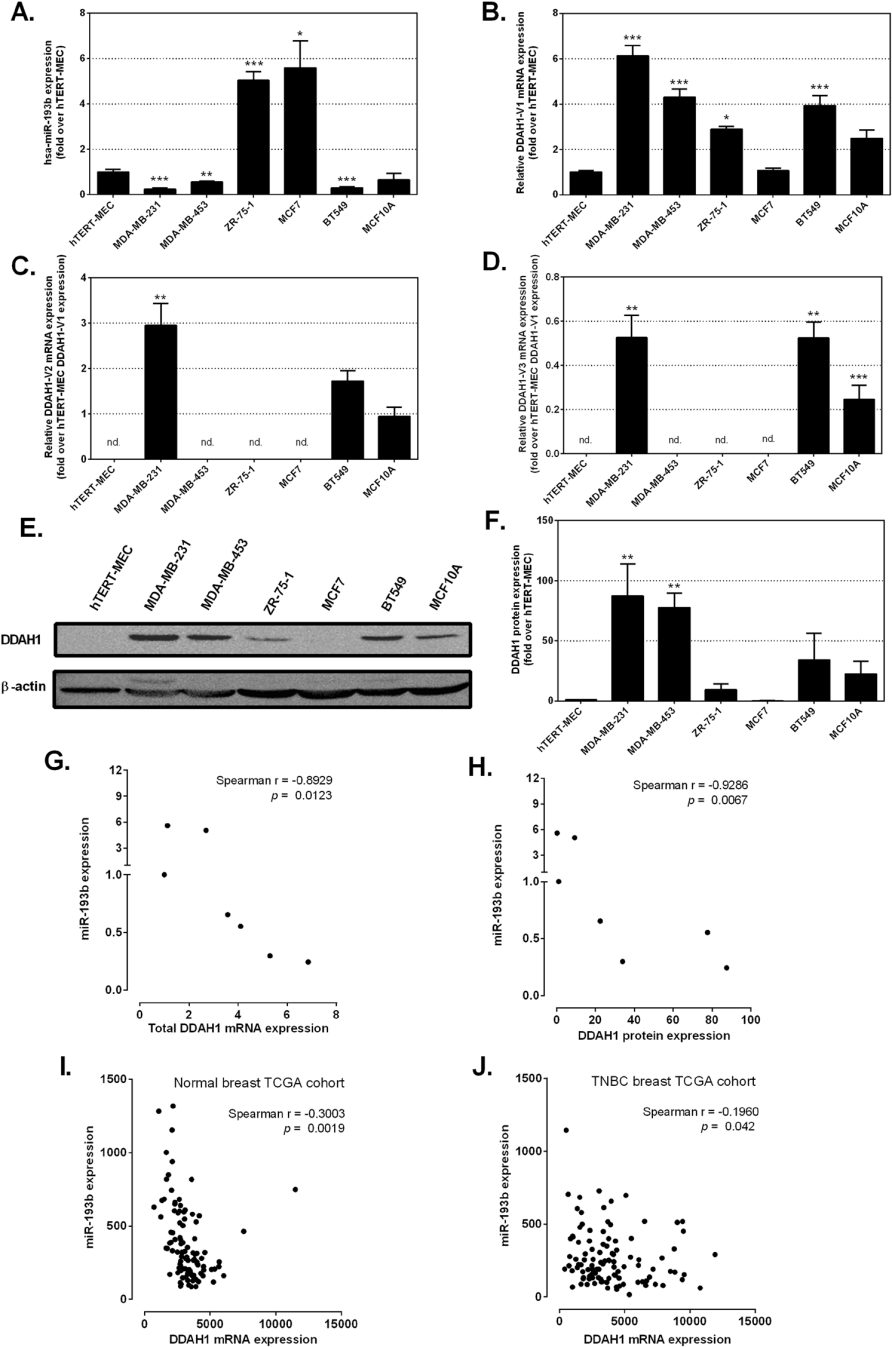
DDAH1 and miR-193b expression is negatively correlated in breast cancer cell lines. (**A**,**B**,**C**,**D**) Expression levels of miR-193b (**A**), *DDAH1-V1* (**B**), *DDAH1-V2* (**C**) and *DDAH1-V3* (**D**) in breast cancer cell lines as assessed by qRT-PCR. Data are normalised to the housekeeping gene 18S (for *DDAH1* variants) or RNU6-2 (for miR-193b) and presented relative to hTERT-MEC cells, which is set to a value of 1. (**E**) Expression of endogenous DDAH1-V1 (full length protein) in breast cancer cell lines as shown by a representative western blot, with ß-actin as a loading control. (**F**) Quantification of DDAH1 protein expression as in **(E**) from at least two independent experiments, normalised to ß-actin expression and shown relative to DDAH1 expression in hTERT-MEC, set to a value of 1. Error bars represent SEM. *p < 0.05, **p < 0.01 and ***p < 0.001 relative to expression in hTERT-MEC. (**G**,**H**) Spearman’s rank-order correlation analysis shows a significant inverse correlation between mean miR-193b expression and mean total *DDAH1* mRNA (**G**) and protein (**H**) expression. Each data point represents a single cell line. Data are the average of at least two independent experiments performed in triplicate. (**I**,**J**) Spearman’s rank-order correlation analysis of The Cancer Genome Atlas (TCGA)/breast carcinoma (BRCA) dataset shows a significant inverse correlation between DDAH1 and miR-193b in normal breast specimens (**I**) and TNBC specimens (**J**).

We sought to quantify expression of DDAH1 and miR-193b in a selection of breast cancer cell lines. qRT-PCR was performed to measure expression levels in immortalized normal human primary mammary epithelial cells (hTERT-MEC), non-tumorigenic mammary epithelial cells of fibrocystic disease origin (MCF10A) and the breast cancer cell lines MCF7, ZR-75-1, MDA-MB-231, MDA-MB-453 and BT549. We found miR-193b expression in all cell lines, however the level of expression was significantly reduced in the triple-negative MDA-MB-231, MDA-MB-453 and BT549 cells compared to that of hTERT-MEC (Fig. 2A). Interestingly, miR-193b expression was significantly higher in MCF7 and ZR-75-1 cells compared to hTERT-MEC. *DDAH1* mRNA expression was assessed by the use of three transcript variant-specific primer sets to specifically amplify all three reported *DDAH1* variants (*DDAH1-V1*, *DDAH1-V2* and *DDAH1-V3*)^42^. All cell lines expressed mRNA corresponding to the *DDAH1-V1* transcript variant encoding the full-length active DDAH1 protein (Fig. 2B). *DDAH1-V1* transcript expression was significantly higher in MDA-MB-231, BT549, MDA-MB-453 and ZR-75-1 cells compared to hTERT-MEC. In addition, *DDAH1-V2* and *DDAH1-V3* transcripts were expressed in MDA-MB-231, BT549 and MCF10A cells (Fig. 2C and D). There was no detectable *DDAH-V2* or *-V3* transcript expression in hTERT-MEC, MDA-MB-453, MCF7 or ZR-75-1 cells. When assessed at the protein level, DDAH1 expression was not detectable in hTERT-MEC and MCF7 cells despite the abundant mRNA expression. DDAH1 protein was most highly expressed in MDA-MB-231, BT549 and MDA-MB-453 cells, but also expressed in ZR-75-1 and MCF10A cells (Fig. 2E and F). The anti-DDAH1 antibody used in this study recognises the epitope TCCSVLINKKVDS, corresponding to amino acids 272–284 of full length hDDAH1. This peptide region is also present in the truncated protein encoded by the DDAH1-V2 and -V3 transcript variants. However, no expression of the truncated DDAH1 protein was detected in the cell lines tested (data not shown).

A Spearman’s rank correlation analysis for all cell lines assessed showed a significant inverse correlation between miR-193b and DDAH1 expression at both the transcript and protein level (Fig. 2G and H). We further extended this study by analysing the TCGA-BRCA RNASeq dataset that contained 104 normal breast and 1,084 breast carcinoma specimens. When all 1,188 samples were analysed together there was no correlation between DDAH1 and miR-193b expression (Supplementary Figure 1A). However, analysis of only the normal breast specimens (104) or breast carcinomas of the triple negative subtype (109 specimens) revealed a significant negative correlation (Fig. 2I and J). Furthermore, analysis of the TCGA-COAD RNASeq dataset (461 colorectal adenocarcinoma specimens) also revealed a significant negative correlation between DDAH1 and miR-193b (Supplementary Figure 1B).

### miR-193b expression reduces DDAH1 mRNA, protein and L-citrulline formation in MDA-MB-231 cells

**Figure 3:**
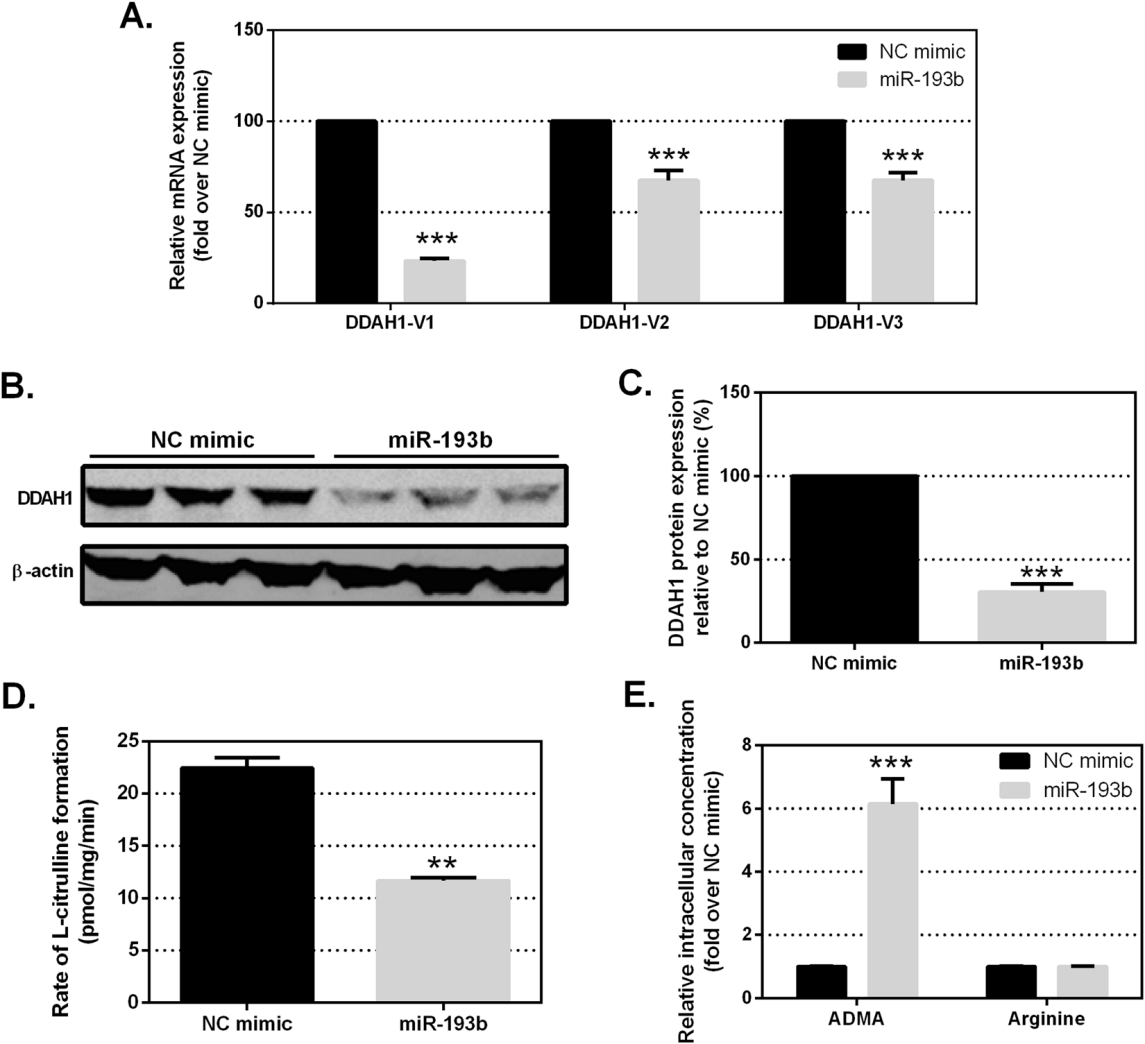
Expression of a miR-193b mimic reduces DDAH1 mRNA, protein and metabolic activity in MDA-MB-231 cells. (**A**) Expression levels of *DDAH1-V1*, *DDAH1-V2* and *DDAH1-V3* mRNA in MDA-MB-231 cells following transfection of 30 nM NC or miR-193b mimic. Expression levels were assessed 48 hr post-transfection by qRT-PCR and normalised to expression of 18S. Expression of *DDAH1* mRNA following miR-193b transfection is shown relative to NC mimic transfection, set to a value of 100%. Data are average of two independent experiments performed in triplicate. (**B**) DDAH1 protein expression was assessed by western blotting 48 hr post-transfection. ß-actin expression was used as a control for total protein. (**C**) Quantification of DDAH1 protein expression as in (**B**) from two independent experiments, normalised to ß-actin expression and shown relative to DDAH1 expression in NC condition, set to 100%. (**D**) DDAH1 activity in MDA-MB-231 cells transfected with 30 nM NC or miR-193b mimic for 48 hr. The activity of DDAH1 in cell lysates is reported as the rate of L-citrulline formation with 200 µM ADMA as substrate. (**E**) Relative concentrations of intracellular ADMA and arginine quantified in MDA-MB-231 cells transfected with 30 nM NC or miR-193b mimic for 48 hr. Error bars represent SEM. **p < 0.01 and ***p < 0.001.

To investigate the potential negative regulation of DDAH1 by miR-193b in breast cancer cells we transfected a miR-193b mimic into the MDA-MB-231 cell line. This cell line was chosen as a model of high endogenous DDAH1 expression and low miR-193b expression (see Fig. 2). Transfection of a miR-193b mimic (30 nM) resulted in a significant reduction in *DDAH1-V1* transcript expression compared to cells transfected with the non-targeting NC mimic (Fig. 3A). Similarly, expression of *DDAH1-V2* and *-V3* transcripts was significantly reduced, although not to the same extent as *DDAH1-V1* (Fig. 3A). Western blotting for DDAH1 protein also revealed a significant reduction following over-expression of miR-193b compared to NC mimic-transfected cells (Fig. 3B and C). Transfection of the miR-193b mimic at a range of concentrations commonly used in the literature^43,44^ revealed a dose-dependent response with 30 nM as the optimal concentration for inhibition of DDAH1 expression. However, concentrations as low as 1 nM were sufficient to significantly inhibit DDAH1 expression (Supplementary Figure 2).

We then assessed activity of DDAH1 following transfection of miR-193b or NC mimic. Consistent with the observed reduction in DDAH1 protein expression, ADMA conversion to L-citrulline was significantly reduced (~50%) upon transfection of miR-193b, as quantified by UPLC-MS (Fig. 3D). Linearity of L-citrulline formation, with respect to incubation time and MDA-MB-231 cell protein concentration, was assessed for assay optimisation and reproducibility (see Supplementary Figure 3). In these samples we also observed a significant accumulation of intracellular ADMA, but not arginine, in cells transfected with miR-193b, consistent with a reduction of DDAH1 protein (Fig. 3E).

### Inhibition of miR-193b elevates DDAH1 mRNA and protein in MCF7 cells

**Figure 4:**
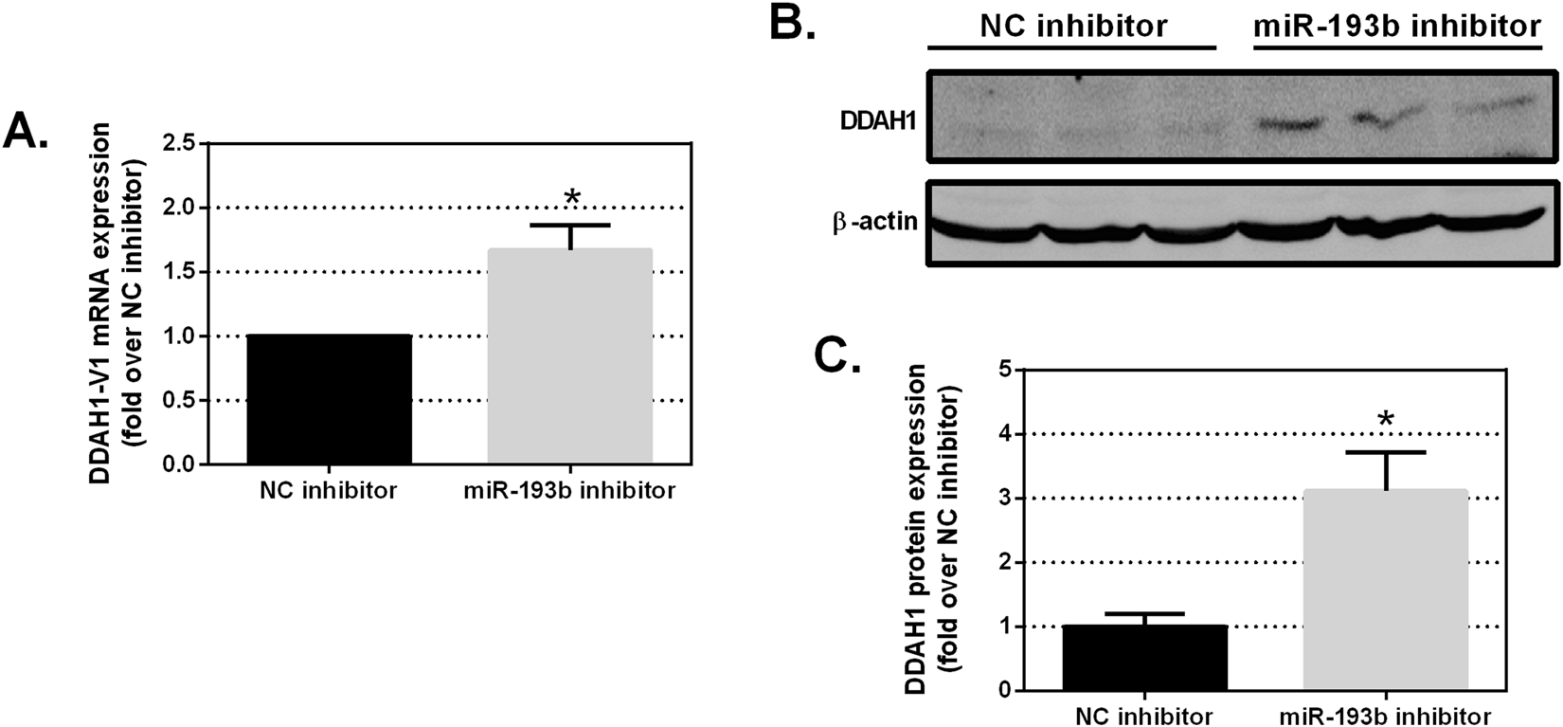
Inhibition of endogenous miR-193b increases DDAH1 mRNA and protein expression in MCF7 cells. (**A**) *DDAH1* mRNA expression levels were assessed following transfection of 100 nM control inhibitor or targeting miR-193b inhibitor. Expression levels were assessed 48 hr post-transfection by quantitative RT-PCR and normalised to expression of 18S. Expression of *DDAH1* mRNA following miR-193b inhibitor transfection is expressed relative to the NC inhibitor transfection, set to a value of 1. (**B**) DDAH1 protein expression was assessed by western blotting 72 hr post-transfection. ß-actin expression was used to normalise for total protein. (**C**) Quantification of DDAH1 protein expression was conducted from three independent experiments and normalised to ß-actin expression. Data are shown relative to DDAH1 expression in NC condition, which is set to a value of 1. All data are the average of at least two independent experiments performed in triplicate. Error bars represent SEM. **p < 0.01 and ***p < 0.001.

MCF7 cells were chosen for miR-193b inhibition experiments due to their high endogenous expression of miR-193b and absence of DDAH1 protein expression. MCF7 cells were transfected with miR-193b or NC inhibitors and DDAH1 expression was assessed 72 hr post-transfection. When compared to the NC inhibitor, transfection of the miR-193b inhibitor significantly increased *DDAH1-V1* mRNA transcript expression (Fig. 4A). Furthermore, inhibition of miR-193b expression resulted in an induction in DDAH1 protein expression. Whilst DDAH1 protein was absent in MCF7 cells transfected with a NC inhibitor, it was detectable at low levels following miR-193b inhibition (Fig. 4B and C). Although DDAH1 protein was detectable by western blot analysis following inhibition of miR-193b, total DDAH1 protein remained too low to enable quantification of DDAH1 metabolic activity (data not shown).

### The 3′UTR of DDAH1 is a direct target of miR-193b

**Figure 5:**
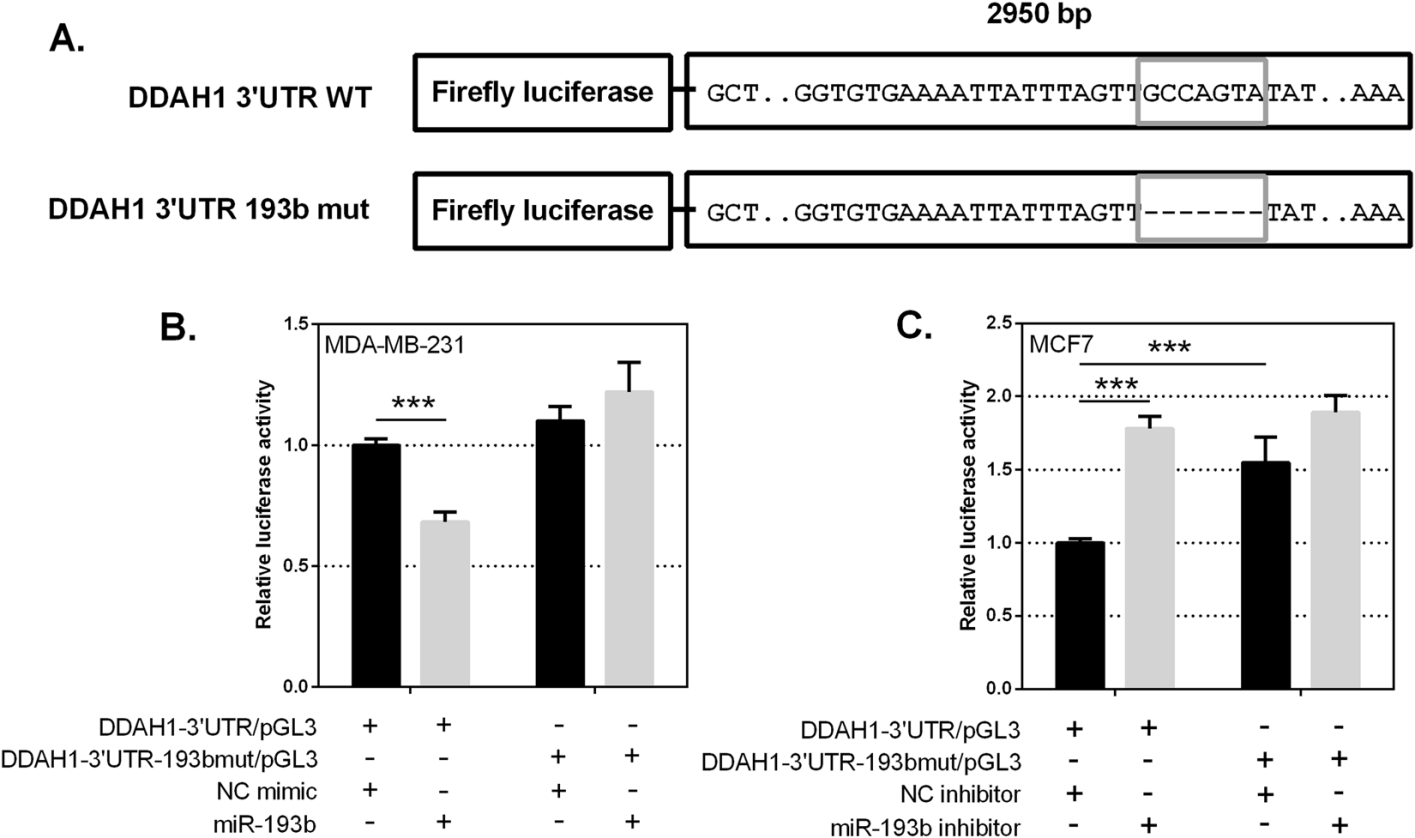
The 3′UTR of *DDAH1* is a direct target of miR-193b in MDA-MB-231 and MCF7 cells. Constructs carrying the WT *DDAH1* 3′UTR or a *DDAH1* 3′UTR with a deleted miR-193b seed site downstream of a firefly luciferase gene were generated. (**A**) Schematic representation of *DDAH1* 3′UTR luciferase reporter constructs with WT (*DDAH1* 3′UTR WT) or deleted miR-193b seed site (*DDAH1* 3′UTR 193b mut). The putative miR-193b seed site is boxed in grey. (**B**) Luciferase analyses in MDA-MB-231 cells. Co-transfection of 30 nM miR-193b mimic reduces *DDAH1* 3′UTR reporter activity, but not upon deletion of the miR-193b seed site. (**C**) Luciferase analyses in MCF7 cells. Co-transfection of 100 nM miR-193b inhibitor increases *DDAH1* 3′UTR reporter activity. Deletion of the miR-193b seed site increases basal reporter activity and prevents further activation mediated by miR-193b inhibitors. Luciferase activities were measured 24 hr post transfection and are expressed as the mean *firefly/Renilla* luciferase ratio relative to that of empty pGL3-promoter vector and normalised to NC mimic or inhibitor transfection, set to a value of 1. Data are the average of at least two independent experiments performed in quadruplicate. Error bars represent SEM. ***p < 0.001.

A luciferase assay system was used to determine whether *DDAH1* is a direct target of miR-193b. Reporter plasmids were constructed to contain either an intact or deleted putative miR-193b binding site (Fig. 5A). MDA-MB-231 cells were co-transfected with each construct in combination with miR-193b or NC mimics, and Firefly and *Renilla* luciferase activities were quantified after 24 hr. A decrease in normalised luciferase activity was used as an indicator of miR-193b binding and thus repression. Cells transfected with DDAH1-3′UTR/pGL3 showed significantly reduced luciferase activity when co-transfected with miR-193b mimic relative to NC mimic (Fig. 5B). This reduction was abolished and luciferase activity was fully restored upon deletion of the miR-193b target site (DDAH1-3′UTR-193bmut/pGL3). With deletion of the miR-193b target site, luciferase activity was similar regardless of transfection with miR-193b or NC (Fig. 5B). A similar result, with a greater repression of DDAH1-3′UTR/pGL3 following transfection with 193b-3p mimic, was observed in HEK293T cells (Supplementary Figure 4).

To complement these findings, we also assessed interaction of miR-193b with the *DDAH1* 3′UTR in MCF7 cells. Co-transfection of DDAH1-3′UTR/pGL3 with miR-193b inhibitor showed a significant increase in luciferase activity compared to transfection with NC inhibitor control (Fig. 5C). Deletion of the miR-193b binding site increased basal activity of the *DDAH1* 3′UTR and abolished the miR-193b inhibitor-mediated increase. Taken together, these results demonstrate the functional importance of the miR-193b binding site within the *DDAH1* 3′UTR and provide evidence that *DDAH1* is a direct target for miR-193b in breast cancer cells.

### DDAH1 regulates tube formation by breast cancer cells

**Figure 6:**
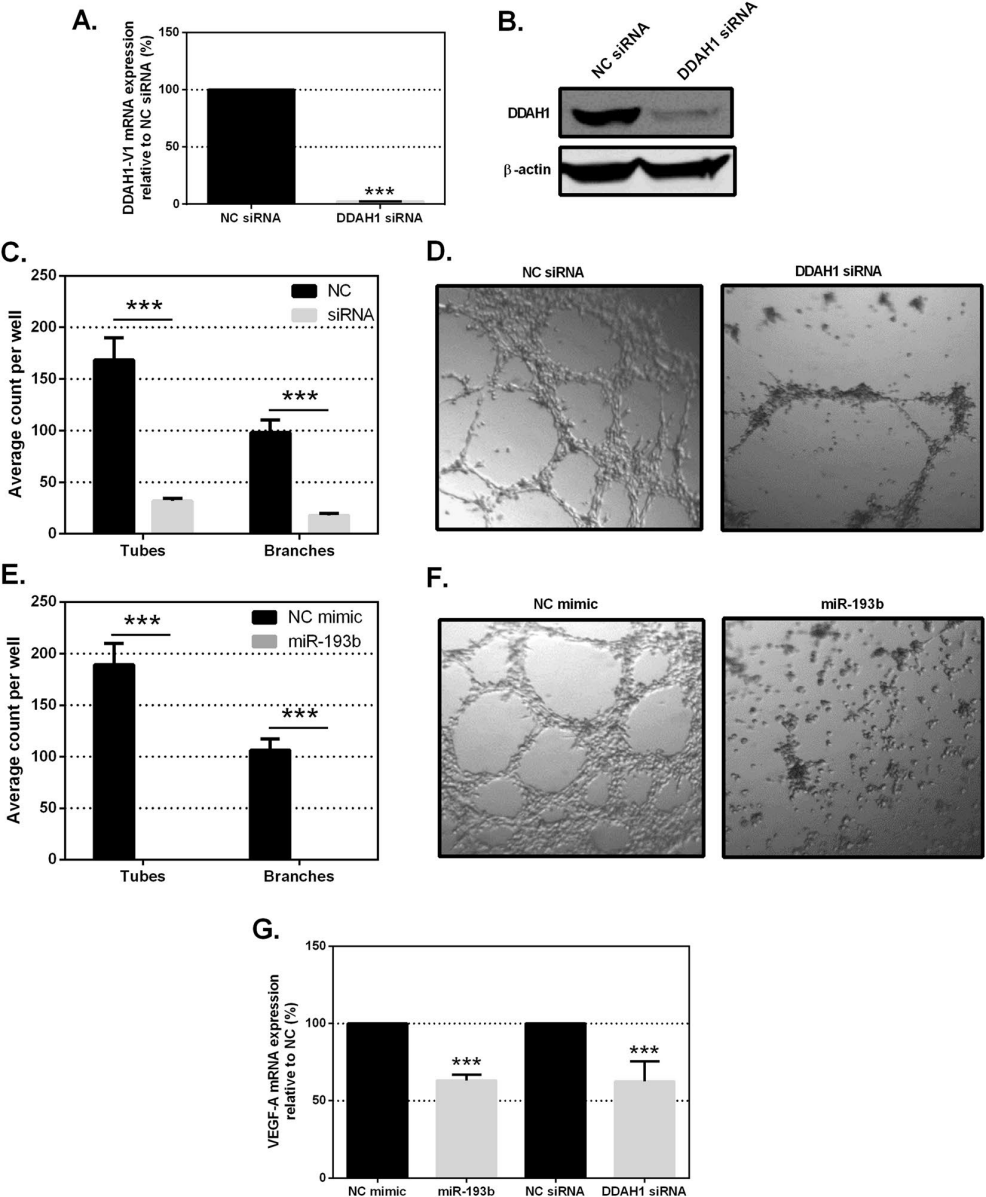
DDAH1 regulates the formation of tube-like structures by MDA-MB-231 cells on Matrigel. (**A**,**B**) Validation of DDAH1 gene knockdown following transfection with 30 nM DDAH1-targeting or control siRNA for 48 hr. *DDAH1-V1* mRNA expression was normalised to 18S expression and is shown relative to control siRNA transfection, set to 100% (**A**). DDAH1 protein expression was assessed by western blotting (**B**). (**C**) MDA-MB-231 cells were transfected with 30 nM DDAH1-targeting siRNA or NC and the number of tubes and branches formed per well were quantified 24 hr after seeding on Matrigel. (**D**) Representative phase contrast images. (**E**) MDA-MB-231 cells were transfected with 30 nM miR-193b mimic or NC and the number of tubes and branches formed per well were quantified 24 hr after seeding on Matrigel. (**F**) Representative phase contrast images. Data shown are mean of three independent experiments performed in triplicate. (**G**) Expression of VEGF-A mRNA in MDA-MB-231 cells following transfection of 30 nM miR-193b mimic, DDAH1-targeting siRNA or appropriate controls. Expression was assessed 48 hr post-transfection and normalised to expression of 18S. Data are average of at least two independent experiments. Error bars represent SEM. ***p < 0.001.

The functional significance of DDAH1 expression in breast cancer cells has not been previously documented. Due to an increase in DDAH1 expression among breast cancer cells representing the particularly invasive triple negative subtype, we hypothesised that DDAH1 regulates at least one aspect of breast cancer cell biology that contributes to tumour growth and/or metastasis. Based on our current understanding of DDAH1 expression and function in vascular biology^21^, we hypothesised that a key function for DDAH1 in breast cancer may be the promotion of VM. We therefore used Matrigel-based tube formation assays to determine the vasculogenic activity of MDA-MB-231 cells^45^. MDA-MB-231 cells formed an extensive network of tube-like structures which was assessed after 24 hr of seeding on Matrigel, thus mimicking the behaviour of endothelial cells and demonstrating a high capacity to undertake VM. To determine a role for DDAH1 in regulating this process, gene silencing with siRNAs was performed 48 hr prior to seeding on Matrigel. Efficacy of DDAH1 knockdown was confirmed (Fig. 6A and B). When these cells were seeded onto Matrigel we observed a significant reduction (80%) in the number of tubes and branches formed compared to control-transfected cells (Fig. 6C). Interestingly, DDAH1-knockdown cells either formed fragmented networks, irregularly shaped clusters of cells, or remained as single cells (Fig. 6D). We then assessed tube formation by cells transfected with miR-193b mimic. Strikingly, we observed complete inhibition of tube formation compared to the NC mimic (Fig. 6E). Cells expressing elevated miR-193b remained as single cells or formed dense, irregular clusters (Fig. 6F). In an attempt to identify the mechanism for the reduction in tube formation, we assessed expression of vascular endothelial growth factor A (VEGF-A), a potent inducer of angiogenesis and VM. Following both direct (siRNA) and indirect (miR-193b) inhibition of DDAH1 we found a significant reduction in VEGF-A mRNA (Fig. 6G).

We also assessed the ability of MCF7 cells to form vascular networks. Control transfected MCF7 cells failed to form tubular structures and stable DDAH1 over-expression was not sufficient to induce tube formation. Transfection of miR-193b inhibitor resulted in a change in morphology of MCF7 cells when grown on Matrigel, however their ability to undertake VM was insufficient and no true tubes were formed (Supplementary Figure 5).

### Investigation of DDAH1-mediated tube formation

**Figure 7:**
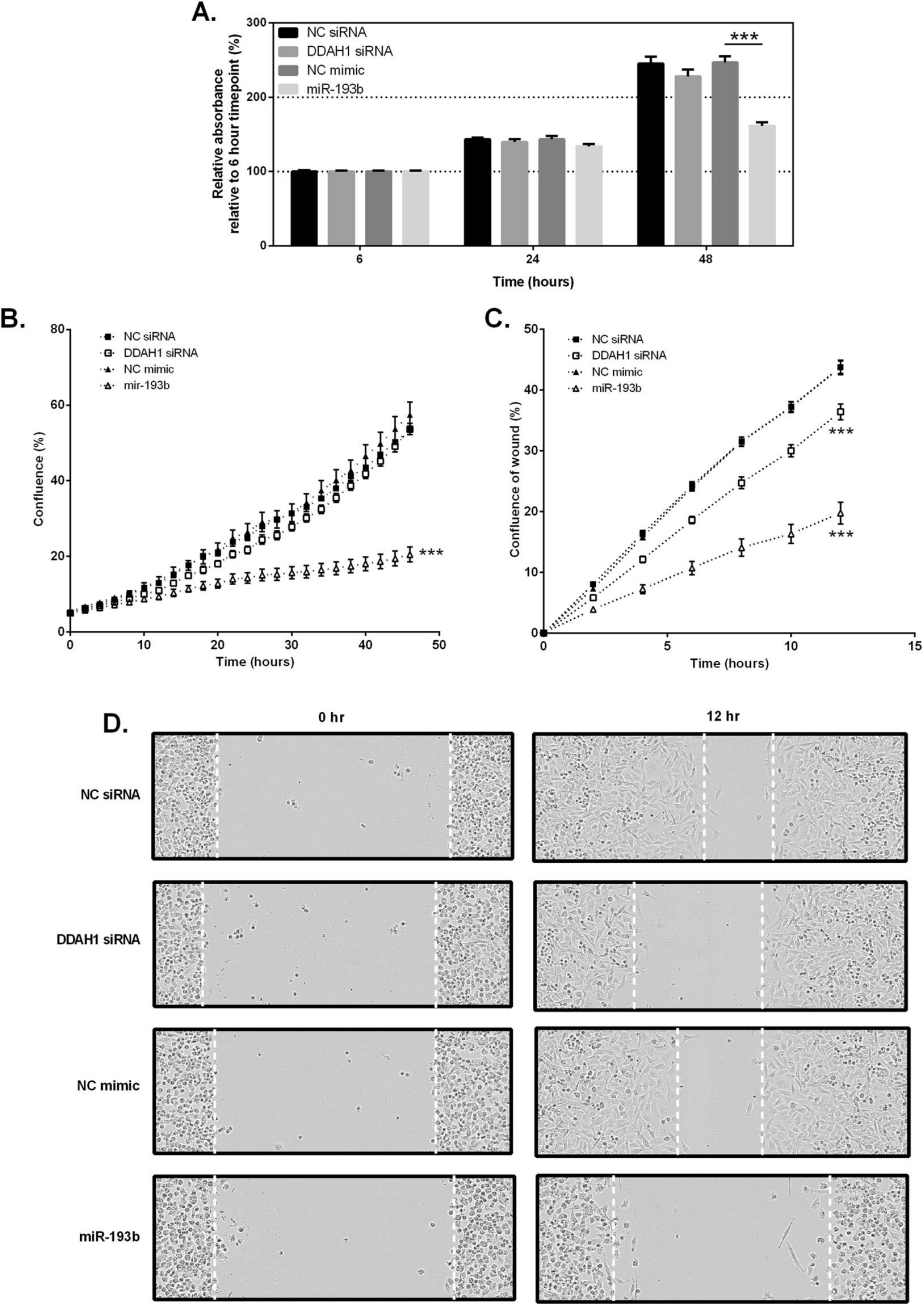
Effect of DDAH1 inhibition on MDA-MB-231 cell proliferation and migration. (**A**) MDA-MB-231 cells were transfected with 30 nM DDAH1-targeting siRNA, miR-193b mimic or appropriate controls and proliferation assessed by staining with crystal violet at 6, 24 and 48 hr post transfection. Absorbance was measured at 570 nm after extensive washing. (**B**) MDA-MB-231 cells were transfected with 30 nM DDAH1-targeting siRNA, miR-193b mimic or appropriate controls and proliferation was assessed using the IncuCyte™ imaging system. Confluence was measured every 2 hr for a 48 hr period. (**C**) Scratch wound migration assays were performed with MDA-MB-231 cells using the IncuCyte™ imaging system following generation of a wound in a confluent cell monolayer. Relative wound density was determined across 3 sections per well over a 12 hr period. Data are represented as the mean of two independent experiments performed in triplicate. Error bars represent SEM. ***p < 0.001 relative to appropriate control. (**D**) Representative images of 0 hr and 12 hr time points for transfected MDA-MB-231 cells quantified in (**C**).

We assessed the effect of DDAH1 inhibition in breast cancer cells across a variety of parameters: cell death, proliferation and migration, to determine whether they could contribute to the observed reduction in tube formation. Knockdown of DDAH1 with siRNA had no significant effect on cell proliferation or change in cell viability over 48 hr as assessed by the trypan blue exclusion assay (data not shown), the crystal violet assay and quantification using the Incucyte™. There was, however, a significant reduction in cell proliferation following expression of miR-193b mimics at 48 hr post-transfection (Fig. 7A and B). We assessed cell migration by means of a scratch wound assay, whereby a uniform scratch was created in confluent cell monolayers. Confluence of the wound was assessed every 2 hr as cells migrated to close the wound. Time-course curves revealed a significant delay in wound closure time for DDAH1-knockdown cells (Fig. 7C). This delay was even more pronounced in cells transfected with miR-193b mimics and was especially apparent by a lack of wound closure (Fig. 7D).

## Discussion

Accounting for approximately 1 in 5 breast cancers, TNBC lacks the three most commonly targeted receptors, ER, PR and HER2, and is characterised by an aggressive and invasive phenotype with poor patient survival due to a lack of effective therapeutic targets^3,46^. With limited treatment options available, insights into the cellular and molecular mechanisms underlying TNBC and the identification of new molecular targets for predicting cancer outcome and intervening in tumour progression, are urgently needed. The present study identifies for the first time that DDAH1 is upregulated at both the mRNA and protein level in numerous breast cancer cell lines. Of particular note, DDAH1 expression was most abundant in the three TNBC cell lines (MDA-MB-231, MDA-MB-453 and BT549) whereas it was absent in normal mammary epithelial cells. In line with these findings, recent evidence suggests DDAH1 is expressed in prostasomes derived from prostate cancer metastases^47^ and is upregulated in metastatic malignant melanoma^48^, whilst over-expression of DDAH1 results in increased tumour growth, vascularization and elevated VEGF secretion *in vivo* ^49,50^.

MicroRNAs function through post-transcriptional regulation of gene expression and play an important role in regulating many physiological and pathological processes including apoptosis, proliferation, migration and differentiation^51^. In particular, dysregulation of miRNAs has been implicated in both initiation and progression of many disease states including cancer. The expression of miR-193b and its correlation with various cancers has been one of particular focus in recent years. Here, we identified a significant reduction in miR-193b expression in three TNBC cell lines compared to non-TNBC cell lines and normal mammary epithelial cells. We have shown that an inverse correlation between miR-193b and DDAH1 expression occurs in multiple breast cancer cell lines. Our analysis of the TCGA-BRCA dataset showed that this correlation also exists in normal breast tissues as well as triple negative breast carcinoma. A similar analysis of the TCGA-COAD dataset also showed an inverse correlation between DDAH1 and miR-193b expression. Whether this relationship extends to any other tissues remains to be investigated. A cohort of matched normal and tumour tissues with defined cancer staging and molecular subtype characterisation may provide further extensive information surrounding miR-193b/DDAH1 expression.

The findings regarding miR-193b expression are concordant with previous reports that identify miR-193b as a key positive regulator of tumour suppression in a range of solid tumours. In breast cancer tissues, miR-193b is downregulated and has previously been reported as a tumour suppressor closely linked with clinical metastasis^39,52^. In addition, miR-193b expression rapidly increases in metformin-treated TNBC cells, which are more sensitive to the effects of metformin than non-TNBC cell lines^53^. miR-193b is also frequently downregulated in other cancers, with studies undertaken in ovarian cancer^54^, melanoma^38^, liver cancer^55^ and prostate cancer^37^. Most recently, increase in miR-193b promoter methylation (consistent with decreased miR-193b expression), as analysed in tissue and urine, has demonstrated a high sensitivity and specificity for prostate cancer detection^56^. In contrast, a small selection of studies describe miR-193b as an oncomiR, and report over-expression of this miRNA in cancers including those of cervical and colorectal origins^57,58^.

The reported contradictory findings in regards to a role for miR-193b in progression of different cancers may be indicative of a multitude of downstream target genes, and whether these genes are activated or suppressed may depend on the specific tumour microenvironment. Due to the observed negative correlation between miR-193b and DDAH1 in breast cancer cell lines within this study, we hypothesised that *DDAH1* is a target of miR-193b. Generally, the extent of miRNA-mediated repression of gene expression is dependent upon the degree and nature of sequence complementarity between the miRNA and the mRNA target^34^. Indeed, bioinformatic analyses of the *DDAH1* 3′UTR revealed a putative binding site for miR-193b with extensive seed pairing and additional 3′-sequence pairing. In turn, this would be expected to highly favour the mRNA degradation/protein translation inhibition pathway. Our data clearly show a reduced mRNA and protein expression of endogenous DDAH1 in MDA-MB-231 cells transiently transfected with a miR-193b mimic, demonstrating the ability of miR-193b to promote both mRNA degradation as well as causing a translational inhibitory effect. Furthermore, by employing a UPLC-MS assay we observed a significant intracellular increase in the concentrations of the DDAH1 substrate, ADMA, as well as a reduction in DDAH1 enzymatic activity as assessed by L-citrulline formation. Conversely, inhibition of miR-193b expression in MCF7 cells was sufficient to induce endogenous DDAH1 expression, and evoked protein expression that was a previously undetectable. Construction of a luciferase reporter plasmid bearing the complete *DDAH1* 3′UTR enabled us to demonstrate that miR-193b decreases reporter activity whilst a miR-193b inhibitor results in increased reporter activity. Deletion of the miR-193b binding site within the *DDAH1* 3′UTR was sufficient to completely abolish these observed effects. Together, these findings suggest that miR-193b inhibits DDAH1 expression directly through targeting is 3′UTR via the miR-193b target site. It is feasible to assume that miR-193b downregulation may enhance the expression of DDAH1 in breast cancer *in vivo*.

For human *DDAH1*, three transcript variants exist and have been previously characterised in endothelial cells: *DDAH1-V1*, *DDAH1-V2* and *DDAH1-V3* ^42,59^. Whilst the *DDAH1-V1* transcript encodes a full length protein, *DDAH1-V2* and *DDAH1-V3* both share a different promoter and encode for an N-terminally truncated protein. Previous analyses of these variants suggest only DDAH1-V1 is responsible for ADMA degradation in human umbilical vein endothelial cells (HUVECs)^42^. To date, the majority of DDAH1 research focuses solely on DDAH1-V1, with our understanding of the regulation and function of DDAH1-V2 and -V3 largely unknown. However, all DDAH1 variants share a common 3′UTR, and the idea that *DDAH1-V2* and *-V3* act as miRNA sponges was identified recently by Kuang and colleagues^59^. We found that in addition to *DDAH1-V1*, *DDAH1-V2* and *-V3* transcripts were also expressed in two of the three TNBC cell lines. However, no protein expression coinciding with these transcripts could be detected. In MDA-MB-231 cells, forced miR-193b expression reduced *DDAH1-V2* and *-V3* mRNA expression in a similar manner to that of *DDAH1-V1*. These findings provide further evidence to support the notion that *DDAH1-V2* and *-V3* transcript variants may play a primary role as miRNA sponges, thus acting as competitors for miR-193b (and other miRNAs) binding to and regulating *DDAH1-V1* (and thus functional DDAH1 protein) expression in a range of cellular models.

The significance of the DDAH/ADMA/NO pathway in angiogenesis is well-documented. In endothelial cells the DDAH/ADMA/NO pathway has a regulatory role necessary for effective angiogenesis which involves the activation, proliferation and migration of endothelial cells. Dysregulation of this pathway is associated with impaired angiogenesis, with at least one study demonstrating a pro-apoptotic role for ADMA in certain endothelial types^60^, and others finding a consistent pro-proliferative role for DDAH1^19,61^. Moreover, endothelial cell migration, alignment and cell-cell adhesion are all inhibited through decreased DDAH expression and function^21,62^. It has been suggested that an upregulation of angiogenesis-related genes may be involved in formation of VM channels^13^. In this study we observed that the highly aggressive DDAH1^+^ MDA-MB-231 cells formed patterned vascular channels *in vitro* whilst the non-aggressive DDAH1^−^ MCF7 cells did not. These findings are in agreement with previous reports^63,64^. We thus hypothesised that DDAH1 may play a regulatory role in breast cancer analogous to that reported in endothelial cells. The ability of MDA-MB-231 cells to form tubes *in vitro* was significantly reduced when DDAH1 expression was silenced by siRNA, yet we saw no induction in tube formation in MCF7 cells stably expressing DDAH1. Thus, we propose that DDAH1 is required, but not sufficient, for VM in breast cancer. Mechanistically, we found that a decrease in DDAH1 expression in MDA-MB-231 cells was accompanied by an increase in endogenous ADMA, a competitive inhibitor of all isoforms of NOS. Whilst we did not detect a change in endogenous arginine, a substrate of NOS, this may be explained by the involvement of arginine in several metabolic pathways^65^; it is possible that a steady-state concentration of arginine is maintained despite disruption of NOS activity. It has been reported that endogenous NO enhances synthesis of VEGF^66,67^, a potent inducer of angiogenesis^68^. We assessed expression of VEGF-A following inhibition of DDAH1 via miR-193b expression or DDAH1-targeting siRNA and found a reduction in VEGF-A mRNA under both conditions. Although we are unable to directly measure NOS activity, we propose that our data showing an accumulation of ADMA, combined with a reduction in VEGF-A expression, provides enough support to the idea that DDAH1 inhibition mediated by miR-193b leads to inhibition of NOS activity and thus an increase in NO and reduction in VEGF, the two key players in angiogenesis and VM. Furthermore, we observed that changes in DDAH1 expression ultimately regulated migration, but not proliferation, of MDA-MB-231 cells. However, the difference observed in migratory potential alone is unlikely to account for the observed reduction in tube formation. Further assessment of other areas required for the complex process of VM formation, such as cell invasion and cell-cell/cell-matrix adhesion, is an area worth pursuing.

In alignment with our findings, inhibition of iNOS in TNBC has demonstrated a profound decrease in cell migration and mammosphere formation^69^. However, a significant problem associated with inhibiting NOS is determining how to target the pathological excess of NO without disrupting the homeostatic balance of NO-mediated processes. Inhibition of excessive DDAH1 expression and/or functional activity in breast cancer cells thus presents a means to indirectly inhibit NOS activity and reduce NO synthesis. Together, these results support our hypothesis that DDAH1 is a necessary factor for productive VM. Recently, alternative roles for DDAH1 independent of the ADMA/NO pathway have surfaced. Glioma xenografts expressing an active-site mutant of DDAH1, incapable of metabolising ADMA, grew faster than *DDAH1*-null xenografts, but not as fast as those expressing WT DDAH1^50^. Interestingly, the generation of an intermediate phenotype proposes an unidentified role for DDAH1 in the modulation of tumour growth. The extent of DDAH1 function in modulating VM in this study via ADMA-dependent or -independent processes is currently unknown. However, by employing mass spectrometry to analyse the formation of L-citrulline from the hydrolysis of ADMA, we were able to determine the catalytic activity of endogenous DDAH1 in MDA-MB-231 cells.

Transfection of MDA-MB-231 cells with a miR-193b mimic resulted in a more potent inhibition of tube formation than inhibition of DDAH1 expression alone; no tubes at all formed over a 24 hr period. Further analysis revealed a significant inhibitory role in both cell proliferation and migration for miR-193b. These data confirm the functional role of miR-193b in breast cancer cells and are consistent with previously published reports in cancer models^52,70,71^. We propose that miR-193b inhibits a network of genes involved in the complex processes of VM and may be key in modulating tumour growth and development. Previously identified targets of miR-193b are limited, but include cyclinD1 and ETS1 in hepatoma cells^55^, uPA, HSP40 and RAB22A in MDA-MB-231 cells^39,52^ and MCL-1 in MCF7 cells^72^. Further research is required to determine if other genes involved in the DDAH/ADMA/NO and associated pathways are also targets for miR-193b in cancer.

In summary, our data show for the first time a high expression of DDAH1 in highly aggressive TNBC cell lines which is inversely correlated with a low expression of the tumour suppressor miR-193b. The over-expression of DDAH1 is driven, at least partly, by the reduced expression of miR-193b as evidenced by reporter analysis, mutagenesis and endogenous gene expression and function analysis. Our data reveal a critical role for miR-193b in VM and demonstrates a mechanistic role for DDAH1 as a novel regulator of VM in breast cancer. Whilst our study provides data to better understand the mechanisms of VM formation in breast cancer, additional studies are warranted to further elucidate the role of DDAH1 in regards to tumour growth and progression, and to translate these findings to improved clinical outcomes. We anticipate that altered expression of miR-193b in breast tissue has a downstream effect on a network of genes (including DDAH1) that are critical for tumour pathology; further analysis of this miRNA-regulatory network is ongoing in our laboratory. Nonetheless, our findings provide a rationale for developing DDAH1 small molecule inhibitors^73^ or for the facilitation of miR-193b/DDAH1/ADMA directed approaches for the prevention and treatment of cancer. Our approach presents a new opportunity to improve patient outcomes in the oncology setting.

## Methods

### miRNA mimics, inhibitors, and siRNA

The nucleotide sequence of human (hsa) miR-193b was obtained from miRbase (www.mirbase.org) as AACUGGCCCUCAAAGUCCCGCU (accession MIMAT0002819). Synthetic double-stranded miRNA mimics of hsa-miR-193b and a negative control miRNA (NC mimic) were purchased from Shanghai GenePharma and transfected at 30 nM. miRNA inhibitors of hsa-miR-193b and a negative control (NC inhibitor) were purchased from Shanghai GenePharma and transfected at 100 nM. Control siRNA and DDAH1-targeting siRNA (sc-105276; Santa Cruz Biotechnology) were transfected at 30 nM.

### Cell line maintenance and transfection

The human breast cancer cell lines MDA-MB-231, MCF7, MDA-MB-453 and ZR-75-1, as well as the human embryonic kidney cell line HEK293T were purchased from the ATCC (www.atcc.org). The human breast cancer cell line BT549, human mammary MCF10A cells and the immortalized human primary mammary epithelial cells, hTERT-MEC, were kindly provided by the laboratory of Dr. Marina Kochetkova at the University of Adelaide, South Australia. MDA-MB-231, MCF7, MDA-MB-453 and HEK293T cells were maintained in Dulbecco’s Modified Eagle Medium (DMEM) supplemented with 10% foetal bovine serum (FBS). BT549 and ZR-75-1 cells were maintained in RPMI 1640 medium supplemented with 10% FBS. MCF10A and hTERT-MEC cells were maintained in serum-free Mammary Epithelial Basal Medium (Lonza). All cell lines were maintained at 37 °C and 5% CO_2_. Cells were reverse-transfected using Lipofectamine 2000 (Thermo Fisher Scientific). For RNA and protein analysis, cells were transfected at a density of 2.5 × 10^5^ cells/well in 6-well plates with 8 µL Lipofectamine 2000 per well.

### RNA isolation and reverse transcriptase quantitative polymerase chain reaction (qRT-PCR)

Total RNA was extracted from cells using TRIzol reagent according to the manufacturer’s instructions (Life Technologies). After DNaseI treatment, cDNA was synthesised using NxGEN M-MuLV reverse transcriptase (Lucigen) and random hexamer primers (NEB). Reverse transcription was carried out for 1 hr at 42 °C in a final volume of 20 µL of RT buffer containing random hexamers, 0.5 mM dNTPs, 40 U of RNase inhibitor and 200 U of reverse transcriptase. Generation of miR-cDNA was performed as previously described^74^. DNaseI-treated RNA was polyadenylated using poly(A) polymerase (NEB) at 37 °C for 30 min to generate a poly(A) tail at the 3′-termini of all miRNAs. The poly(A)-tailed miRNAs were transcribed to cDNA using NxGEN M-MuLV reverse transcriptase with the universal oligo (5′-CAGGTCCAGTTTTTTTTTTTTTTTVN-3′)^74^ substituted for random primers, as described above.

All cDNA was diluted 1:5 in RNase-free water before use in qRT-PCR. PCR reactions were performed using a Corbett RotorGene 3000 instrument (Corbett Research) in a 20 µL reaction containing GoTaq SYBR green PCR master mix (Promega), 2 µL of diluted cDNA sample and 500 nM each specific forward and reverse primer. The PCR amplification conditions for standard cDNA were: initial incubation at 95 °C for 15 min, followed by 40 cycles of 95 °C for 10 sec, 62 °C for 15 sec and 72 °C for 20 sec. Amplification of miRNAs consisted of an initial incubation at 95 °C for 15 min, followed by 40 cycles of 95 °C for 10 sec and 60 °C for 40 sec. A temperature ramp from 60 to 95 °C was used to generate a melt curve at the end of each run. Using the 2^−ΔΔCT^ method^75^, the expression level of *DDAH1* was characterised relative to that of ribosomal 18S as a housekeeping gene and the expression level of miR-193b was determined relative to that of housekeeping miR-U6 small nuclear-2 RNA (*RNU6-2*). Target gene primers and miRNA-specific primers used are listed in Supplementary Table 1.

### Cloning and deletion mutagenesis

The *DDAH1* mRNA (NM_012137.3) contains a 2,971 base pair (bp) 3′UTR. A 2,950 bp portion of the *DDAH1* 3′UTR region was cloned from a commercial human genomic DNA sample (Roche Diagnostic). PCR amplification was performed using Phusion hot-start high-fidelity DNA polymerase (Thermo Fisher Scientific) according to the manufacturer’s instructions. The 3′UTR region was cloned into the *Xba*I restriction site of the pGL3-promoter vector (Promega), immediately downstream of the luciferase coding sequence, to generate the reporter construct DDAH1-3′UTR/pGL3. Deletion of the miR-193b binding seed site within the *DDAH1* 3′UTR was undertaken with primers spanning 30–35 bp on either side of the miR-193b seed site, but excluding the seed site itself. The resulting construct is referred to as DDAH1-3′UTR-193bmut/pGL3. The identities of both constructs were confirmed by DNA sequencing. All cloning and mutagenesis primers are listed in Supplementary Table 1.

### Luciferase reporter assays

Cells were reverse transfected at a density of 2.5 × 10^4^ cells/well in 48-well plates. Cells were transfected in quadruplicate with 8 ng of the internal reference pRL-null and 250 ng luciferase reporter (empty pGL3-promoter, DDAH1-3′UTR/pGL3 or DDAH1-3′UTR-193bmut/pGL3) per well. Twenty-four hours post transfection, cells were harvested and assayed for firefly and *Renilla* luciferase activities using a Dual Luciferase assay kit (Promega) as previously described. Luciferase activities were expressed as the mean firefly/*Renilla* luciferase ratio, relative to that of empty pGL3-promoter.

### Western blot analysis

Cultured cells were lysed in RIPA buffer (50 mM Tris pH 7.3, 150 mM NaCl, 0.1% SDS, 0.5% sodium deoxycholate, 1% NP-40) and further disrupted by passages through a 30 G syringe. Following a 20 min incubation on ice, cellular debris was removed via centrifugation at 13,000 × *g* for 10 min. Total protein concentrations were determined using Bio-Rad Protein Assay reagent (Bio-Rad). Sixty micrograms of total protein was separated by SDS-PAGE and transferred to Trans-blot nitrocellulose membrane (Bio-Rad). Membranes were blocked in 4% (w/v) non-fat dry milk in Tris-buffered saline (TBST) for 90 min, followed by incubation with goat anti-DDAH1 antibody (Abcam; ab2231) for 2 hr. Blots were treated with enhanced SuperSignal West Pico chemiluminescent (ECL) hrP substrate (Thermo Fisher Scientific) and imaged using an ImageQuant LAS 4000 (GE Healthcare Life Sciences). Blotting for beta-actin (Thermo Fisher Scientific; MA5-15739) was used as a protein loading control. Densitometry quantification of protein bands was determined using Multi Gauge Ver2.0 software (FujiFilm).

### Analyses of The Cancer Genome Atlas database

Transcriptome profiling data of RNA sequencing (RNAseq) and miRNA sequencing of breast invasive carcinoma (BRCA) and colon adenocarcinoma (COAD) were downloaded from The Cancer Genome Atlas (TCGA) data portal (https://gdc-portal.nci.nih.gov/). The RNAseq expression data were represented in the form of high-throughput sequencing counts. Genes (protein coding and noncoding) with a mean of less than 10 counts were discarded; the counts of the remaining genes were normalized using the upper quantile normalization method. Correlation analyses between the expression levels of DDAH1 and miR-193b were conducted using the Spearman rank method and plots were drawn using GraphPad PRISM software.

### DDAH1 activity assay: Measurement of L-citrulline formation

For assessment of DDAH1 activity *in vitro*, cultured cells were lysed in 100 mM phosphate buffer (pH 7.4) by sonication at 16 × 1 sec bursts at 40% amplitude (Sonics Vibracell). Reactions for L-citrulline formation were undertaken in glass tubes at 37 °C in a 100 µL volume comprising 100 mM phosphate buffer (pH 7.4), MDA-MB-231 cell lysate (100 µg) and ADMA as substrate (0 or 200 μM). Reactions were initiated by the addition of ADMA, and were terminated after 3 hr by the addition of 10 µL L-citrulline-d6 (30 µM assay internal standard) and 300 µl 0.1% formic acid in isopropanol. The reaction mixture was vortex mixed, cooled on ice and centrifuged at 18,000 × *g* for 5 min. A 300 µL aliquot of supernatant was transferred into a glass tube and evaporated to dryness in a MiVac (50 °C, -OH programme, 20 min) (SP Scientific). Samples were reconstituted in 125 μL of 1:4 water/0.1% formic acid in isopropanol and a 3 μL aliquot assayed by UPLC-MS. A detailed description of the assay conditions, including the measurement of ADMA and arginine concentrations, are provided as Supplementary Information.

### Matrigel tube formation assays

Cells were reverse-transfected as previously described. Matrigel® (Corning) aliquots were thawed overnight on ice and 45 µL per well used to coat wells of a 96-well plate. The Matrigel layer was allowed to gel by incubating the plate at 37 °C for 30 min. Forty-eight hours post transfection, cells were resuspended and seeded at 2.5 × 10^4^ cells/well in a total volume of 150 µL in Matrigel-coated wells. Tube formation was assessed after 24 hr. Wells were imaged with an EVOS® FL imaging system (Life Technologies) using a 2× objective. Four images per well were stitched together and the total number of tubes and branches counted using ImageJ software (NIH). Tube-like structures were defined as elongated multi-cellular structures and branches defined as the intersecting points of two or more tubes.

### Cell viability and proliferation

Cell viability was assessed using the trypan blue exclusion assay. Briefly, transfected cells were collected 48 hr post-transfection, resuspended in media and diluted 1:2 with trypan blue. For assessment of cell viability and proliferation by the crystal violet assay and the IncuCyte™ monitoring system (Essen Bioscience), transfected cells were resuspended at 24 hr post-transfection, counted and re-plated. A seeding density of 2.5 × 10^4^ cells/well in 24-well plates was used for the IncuCyte^TM^. Cell confluence was measured across 9 images per well, every 2 hr for a 45 hr period. For the crystal violet assay, cells were seeded at 1.25 × 10^4^ cells/well in 96-well plates. At 6, 24 and 48 hr post seeding, media was aspirated and cells stained with 50 µL 0.5% (w/v) crystal violet in 50% (v/v) methanol per well for 10 min. Plates were rinsed with water to remove non-adherent cells and excess dye, then air-dried overnight. Once dried, 50 µL 33% (v/v) acetic acid was added to each well and incubated for 10 min. Optical density (OD) was measured at 570 nm using a VersaMax microplate reader (Molecular Devices) and analysed with SoftMax Pro software (Molecular Devices).

### Scratch wound migration assays

Confluent cells were serum starved for 4 hr prior to wound generation. Wounds were created in confluent monolayers of cells in a 24-well ImageLock (Essen Bioscience) plate using a WoundMaker™ (Essen Bioscience). Cells were washed twice to remove debris and finally placed in DMEM supplemented with 0.5% FBS. An IncuCyte^TM^ was used to take 3 images per well, every 2 hr for a 12 hr period. Closure of the wound area was assessed by the relative wound density (%) automatic analysis metric of the IncuCyte^TM^, which calculates cell density in the wound area expressed relative to the cell density outside of the wound area over time.

### Statistical analysis

Data are shown as mean ± SEM. Statistical analysis between groups was performed using an independent two-tailed Student’s *t* test, one-way ANOVA or two-way ANOVA as appropriate. Correlations between DDAH1 and miR-193b expressions were analysed using two-tailed Spearman rank correlation analysis. All analyses were performed using GraphPad Prism 6 (GraphPad Software). A p value of less than 0.05 was considered to be statistically significant.

## Electronic supplementary material

Supplementary information
