# Supplementary material for: MiR-193b regulates breast cancer cell migration and vasculogenic mimicry by targeting dimethylarginine dimethylaminohydrolase 1
Source: Sci Rep. 2017 Oct 25;7:13996. doi: 10.1038/s41598-017-14454-1 (PMC5656623; doi:10.1038/s41598-017-14454-1)
Supplement: Supplementary file 1 — Supplementary information [file 41598_2017_14454_MOESM1_ESM.pdf]

MiR-193b regulates breast cancer cell migration and vasculogenic mimicry by targeting dimethylarginine dimethylaminohydrolase 1

Julie-Ann Hulin*[1,2], Sara Tommasi[1], David Elliot[1], Dong Gui Hu[1,2], Benjamin C. Lewis[1,2] and Arduino A. Mangoni[1]

[1]Clinical Pharmacology, Flinders University College of Medicine and Public Health, Flinders Medical Centre, Bedford Park, South Australia, Australia

[2]Flinders Centre for Innovation in Cancer, Bedford Park, South Australia, Australia

*Corresponding author. Phone: (08) 8204 5096. Email: julieann.hulin@flinders.edu.au

Supplementary material

UPLC-MS analysis of L-Citrulline

Citrulline, ADMA and arginine analysis was performed on a Waters ACQUITY™ Ultra Performance LC™ system coupled to a Waters Premier quadrapole time of flight (qToF) mass spectrometer with an electrospray ionisation source operated in positive ionisation mode. Time-of-flight (ToF) data were collected in MS mode between 100 and 500 Da with an instrument scan time of 0.5 sec and inter-scan delay of 0.05 sec. Experimental parameters were as follows: capillary voltage 3.5 kV, source temperature 90°C, desolvation temperature 300°C, sampling and extraction cone voltages were 20 and 5 eV, respectively. The collision gas flow was 0.4 mL/min. Instrument control, data acquisition and data processing were performed using Waters MassLynx version 4.1 software.

Chromatographic separation was performed at a flow rate of 0.3 mL/min on a Waters ACQUITY UPLC® BEH HILIC column (1.7 µm, 2.1 mm x 100 mm) held at 35°C. Mobile phase composition was 0.1% v/v formic acid in acetonitrile (mobile phase A) and 10% v/v acetonitrile in water containing 0.1% v/v formic acid (mobile phase B). Initial conditions were 95% mobile phase A and 5% mobile phase B. The proportion of mobile phase B was increased linearly to 40% over 5 min and then returned to 5% for 2 min to re-establish equilibrium before injection of the following sample for analysis.

Extracted ion chromatograms (EICs) were obtained with a mass window of 0.02 Da from total ion chromatograms (TIC) employing the m/z corresponding to the 176.10→159.10 and 181.13→165.12 fragments of L-citrulline and L-citrulline-d6 respectively. For quantitation, L-citrulline was spiked into MDA-MB-231 lysate at 6 concentrations (0, 1, 2, 3, 4 and 5 µM) then extracted and reconstituted in the same manner as incubation samples. A calibration curve was constructed by plotting the peak area ratio L-citrulline to internal standard versus the L-citrulline concentration (Supplementary Figure 3). Extracted ion chromatograms at m/z

203.15 and 175.13 corresponding to the parent ions for ADMA and arginine were used to assess the relative change in the concentrations of these analytes based on peak area.

Supplementary Table 1. Primers used for qRT-PCR, cloning and mutagenesis. Underline indicates restriction site. / indicates deleted binding site. V = G, C or A but not T. N = G, C, A or T.

**Primer**	**Sequence (5'-3')**
**qRT-PCR**	
18S F	CGATGCTCTTAGCTGAGTGT
18S R	GGTCCAAGAATTTCACCTCT
DDAH1-V1 F	CTTCCGGACTGCGTCTTC
DDAH1-V1 R	TGCTTCTTTCATCATGTCAACC
DDAH-V2 F	CTCAAGTCCTGCCCAAAGAC
DDAH-V2 R	CCTTTTCCATACAGATGAAACCA
DDAH1-V3 F	GCAAATGTTTATGGGTTGACAT
DDAH-V3 R	GCACCTCGTTGATTTGTCCT
VEGF-A F	TGCTGTCTTGGGTGCATT
VEGF-A R	GTGCTGTAGGAAGCTCATCTC
Universal R oligo for miR-cDNA generation	CAGGTCCAGTTTTTTTTTTTTTTTVN
miR-193b F	AACTGGCCCTCAAAGTCC
miR-193b R	GGTCCAGTTTTTTTTTTTTTTAGCG
RNU6-2 F	CGCAAGGATGACACGCA
RNU6-2 R	AAAAATATGGAACGCTTCAC
**Cloning and site-directed mutagenesis**	
DDAH1 3'UTR F	ACTCTAGACGGTAGCCGGCAAGACC
DDAH1 3'UTR R	GCGTCTAGACACAGTACAACTCAACAC
DDAH1 3'UTR 193bmut Top	GGTGTGAAAATTATTTAGTT/TATTCTGAAGAATGTCTTCTTGATCAGTC
DDAH1 3'UTR 193bmut Bottom	GACTGATCAAGAAGACATTCTTCAGAATA/AACTAAATAATTTTCACACC

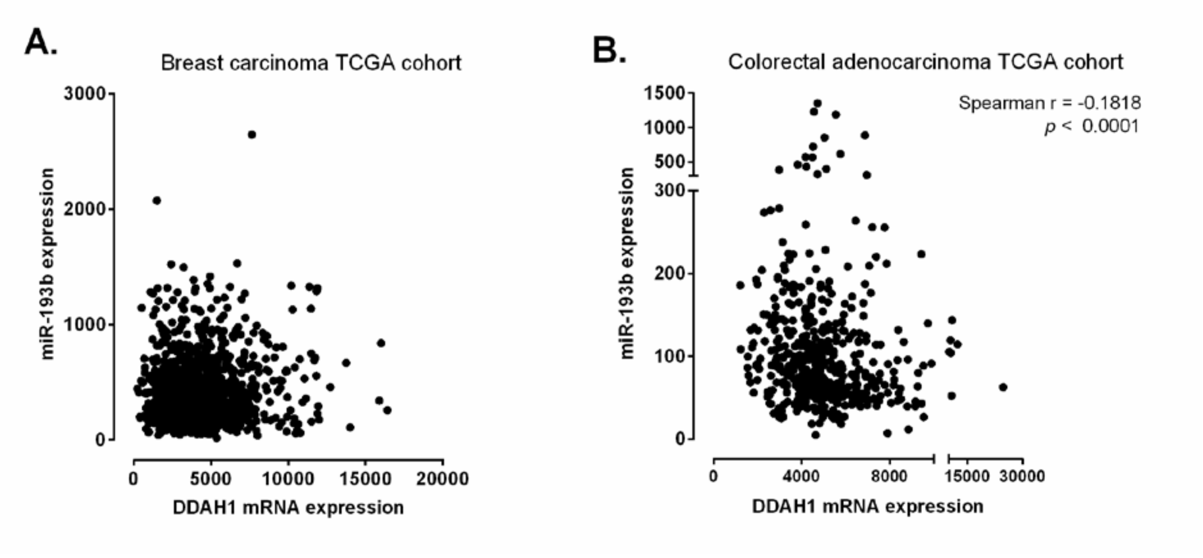

Supplementary Figure 1. Analysis of DDAH1 and miR-193b expression in breast carcinoma and colorectal carcinoma specimens from The Cancer Genome Atlas (TCGA) database. **(A):** Spearman's rank-order correlation analysis shows no correlation between DDAH1 and miR-193b in the breast carcinoma cohort when all 1188 samples including 104 normal breast specimens and 1084 breast carcinoma specimens were analysed together. **(B):** A significant inverse correlation between DDAH1 and miR-193b expression is observed in the colorectal adenocarcinoma cohort that contained 461 cancer specimens.

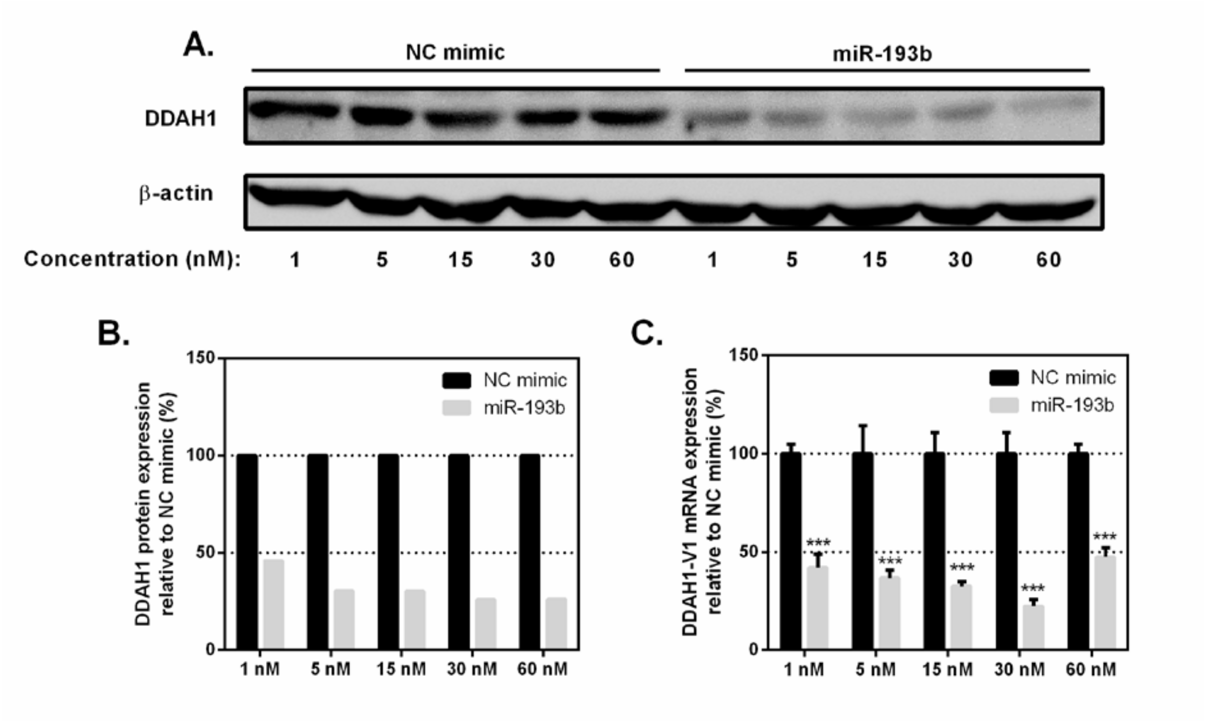

Supplementary Figure 2. Dose-dependent effect of miR-193b on DDAH1 expression in MDA-MB-231 cells. **(A):** Expression of endogenous DDAH1-V1 (full length protein) following transfection of increasing concentrations of miR-193b mimic or control (NC), with ß-actin as a loading control. **(B):** Quantification of DDAH1 protein expression in (A), normalised to ß-actin expression and shown relative to DDAH1 expression in control transfected cells, set to 100%. **(C):** Expression of DDAH1-V1 mRNA as assessed by qRT-PCR. Data are normalised to the housekeeping gene 18S and presented relative to DDAH1 expression in control transfected cells, set to 100%. Error bars represent SEM.

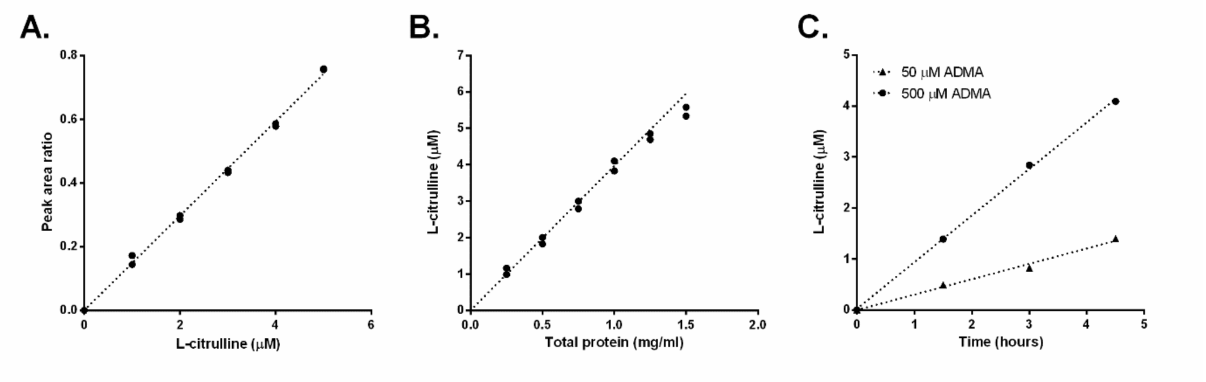

Supplementary Figure 3. Optimisation of DDAH1 activity assay. **(A):** Representative L-citrulline calibration curve. L-citrulline concentrations for matrix-matched calibrators were between 0 and 5 µM and samples were run in duplicate. **(B):** Protein linearity data were collected in duplicate at 200 µM ADMA with a 3 hr incubation period. **(C):** Time linearity was assessed with 50 and 500 µM ADMA with 1 mg/ml total protein.

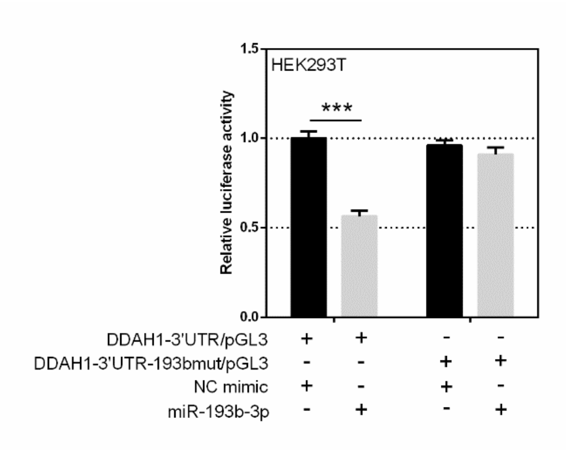

Supplementary Figure 4. The 3'UTR of *DDAH1* is a direct target of miR-193b in HEK293T cells. Constructs carrying the WT *DDAH1* 3'UTR or a *DDAH1* 3'UTR with a deleted miR-193b seed site downstream of a firefly luciferase gene were generated. Co-transfection of 30 nM miR-193b mimic reduces *DDAH1* 3'UTR reporter activity, but not upon deletion of the miR-193b seed site. Luciferase activities were measured 24 hr post transfection and are expressed as the mean *firefly/Renilla* luciferase ratio relative to that of empty pGL3-promoter vector and normalised to NC mimic transfection, set to a value of 1.

Data are the average of at least two independent experiments performed in quadruplicate. Error bars represent SEM. *** p<0.001.

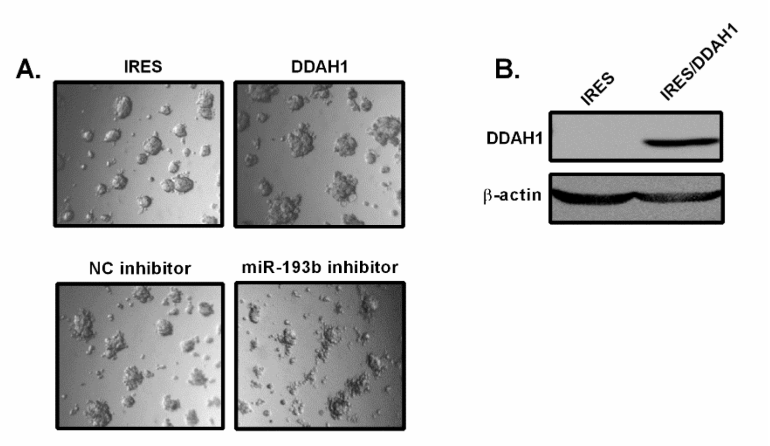

Supplementary Figure 5. DDAH1 over-expression or miR-193b inhibition does not promote tube formation in MCF7 Matrigel-based assays. **(A):** MCF7 cells stably expressing either empty IRES plasmid or IRES/DDAH1 plasmid, or MCF7 cells transiently transfected with either 100 nM NC or miR-193b inhibitor, were imaged 24 hr post seeding on Matrigel. Two independent experiments were performed in triplicate. Representative phase-contrast images are shown. **(B):** Validation of DDAH1 expression in the MCF7 DDAH1-stably expressing cell line. DDAH1 protein expression was assessed by western blotting using ß-actin expression as a control for total protein.

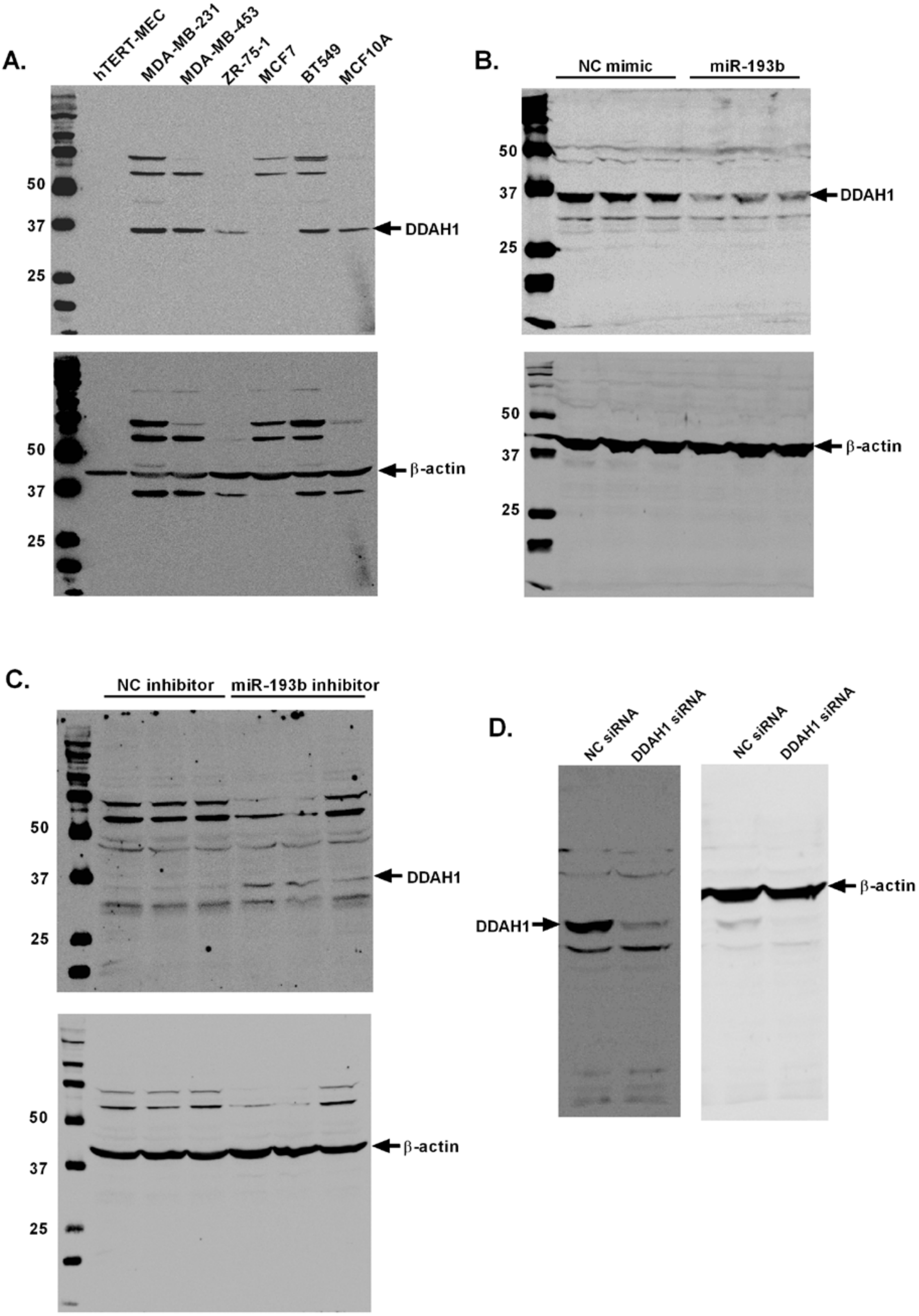

Supplementary Figure 6. Full length images of blots that appear in figures within the main text. **(A):** Blots corresponding to Figure 2E. **(B):** Blots corresponding to Figure 3B. **(C):**

Blots corresponding to Figure 4B. **(D):** Blots corresponding to Figure 6B. DDAH1 (~37 kDa) and ß-actin (~42 kDa) bands are marked. For further experimental details refer to the appropriate legends of the main figures.
